# Is a change in juvenile hormone sensitivity involved in range expansion in an invasive beetle?

**DOI:** 10.1186/s12983-015-0113-1

**Published:** 2015-09-11

**Authors:** Philipp Lehmann, Anne Lyytinen, Saija Piiroinen, Leena Lindström

**Affiliations:** Department of Zoology, University of Stockholm, Stockholm, Sweden; Centre of Excellence in Biological Interactions Research, Department of Biological and Environmental Science, University of Jyväskylä, Jyväskylä, Finland; School of Life Sciences, University of Sussex, Sussex, UK

## Abstract

**Introduction:**

It has been suggested that rapid range expansion could proceed through evolution in the endocrinological machinery controlling life-history switches. Based on this we tested whether the Colorado potato beetle, *Leptinotarsa decemlineata*, which has rapidly expanded its range across latitudinal regions in Europe, and shows photoperiodic adaptation in overwintering initiation, has different sensitivities to juvenile hormone (JH) manipulation along a latitudinal gradient.

**Results:**

A factorial experiment where beetles were reared either under a long or short day photoperiod was performed. Hormone levels were manipulated by topical applications. An allatostatin mimic, H17, was used to decrease and a juvenile hormone III analogue, pyriproxyfen, was used to increase the hormone levels. The effects of photoperiod and hormone manipulations on fecundity and overwintering related burrowing were monitored. Application of H17 decreased fecundity but did not induce overwintering related burrowing. Manipulation with pyriproxyfen increased fecundity and delayed burrowing. While small population-dependent differences in responsiveness to the topical application treatments were observed in fecundity, none were seen in overwintering related burrowing.

**Conclusions:**

The results indicate that the rapid photoperiodic adaptation manifested in several life-history and physiological traits in *L. decemlineata* in Europe is unlikely a result of population dependent differences in JH III sensitivity. While other endocrine factors cannot be ruled out, more likely mechanisms could be genetic changes in upstream elements, such as the photoperiodic clock or the insulin signaling pathway.

## Introduction

Rapid evolution of life-history traits may be required for species to survive large environmental changes arising from range expansion, anthropogenic impact or climate change, as it has been documented repeatedly [1–3]. Rapid life-history evolution could proceed through changes in the endocrinological machinery controlling life-history switches [4–6]. This is because hormones coordinate cascades of downstream molecular and physiological changes [4].

Invasive species provide excellent study systems to investigate how species can respond to large environmental changes [7]. For instance, during range expansion towards high latitudes, many invasive species have synchronized life-history and stress-tolerance traits with local season length and seasonal abiotic fluctuations [1]. A common feature of phenological synchronization is the photoperiodic timing of winter resting stages, such as diapause [8], which generally are triggered by the shortening of photoperiod associated with late summer [9]. The mechanisms by which the photoperiod leads to the diapausing phenotype have been much debated [9, 10]. It is clear that the photoperiodic information at some stage affects endocrinological glands whose hormonal products [11] act as main drivers in the development of the diapausing phenotype [12, 13]. In species which overwinter as adults, such as in the invasive *Leptinotarsa decemlineata*, pre-diapause development is accompanied by decreased Juvenile Hormone III (JH III) titers in the heamolymph, which is due to decreased biosynthesis in the corpora allata and increased degradation by JH-esterases [12, 13]. The inhibition of JH III biosynthesis in the corpora allata is most likely under the control of neurally mediated allatostatins [13, 14].

The rapid range expansion of *L. decemlineata* across Europe is associated with adaptive synchronization of life-history traits with local photoperiodic conditions [9, 12], which could be linked to genetic changes in the endocrinological machinery. This could involve changes in hormone biosynthesis, transport, degradation or hormone receptor function [4, 11, 13]. Here we study population dependent differences in diapause induction in response to negative and positive manipulation of JH III levels. Diapause induction was studied by tracking burrowing behavior and oviposition. Since adult *L. decemlineata* spend winters burrowed in the soil, burrowing behavior is often used as a marker for diapause initiation [12]. Diapause is also a state of reproductive arrest where ovaries are degenerated, and therefore the pre-diapause period is characterized by low oviposition frequency [12]. Both traits are known to be associated with JH III levels [12]. In beetles preparing for diapause, JH III levels should be low [12]. Thus, in these individuals an application of an allatostatin mimic (H17) to decrease JH III levels should have negligible additive effects on fecundity and burrowing [14]. In contrary, increasing JH III levels with a juvenile hormone analog (JHA) should have strong effects on the same traits [15]. In beetles undergoing reproductive development, baseline JH III levels should be high [12]. Therefore, increasing JH III levels should have weak additive effects, while decreasing JH III levels should strongly decrease fecundity and increase burrowing propensity. Differences in receptivity among populations could indicate that adaptation has taken place in the actual endocrinological machinery, as opposed to, for instance, the photoperiodic clock or insulin signaling pathway [4, 5, 10].

## Results

**Fig. 1:**
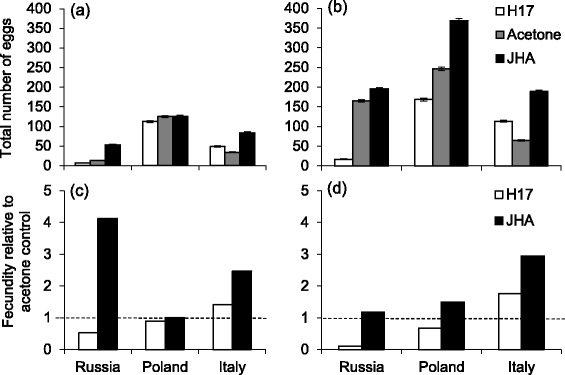
Fecundity of three European *L. decemlineata* populations treated with H17, acetone or JHA and reared in a (a/c) short or (b/d) long photoperiod. **a** and **b** show total egg number while **c** and **d** show egg number relative to the acetone control (no difference = 1). *N* = 16–20 per group. Values are estimated marginal means based on the statistical model including weight as covariate

**Fig. 2:**
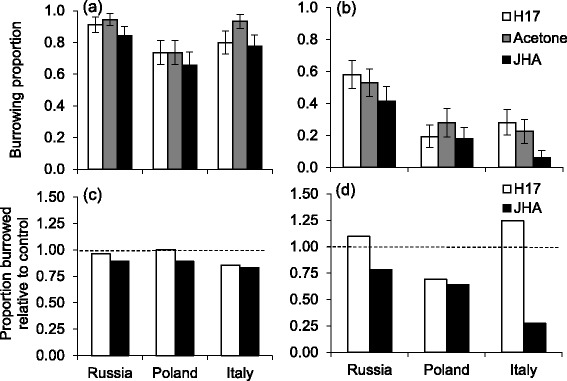
Burrowing proportion of three European *L. decemlineata* populations treated with H17, acetone or JHA and reared in either a (a/c) short or (b/d) long photoperiod. **a** and **b** show absolute burrowing proportion while **c** and **d** show burrowing proportion relative to the acetone control (no difference = 1). *N* = 32–42 per group

The descendants of field-collected beetles from Russia, Poland and Italy were used in the present experiment. From each population, adult beetles were reared under a short (SD: 12 h light) or long (LD: 18 h light) photoperiod, and subjected to topical applications of either acetone, H17 or JHA (see Methods section for details) in a split-brood design. Beetles were then reared in pairs and fecundity as well as burrowing was tracked for 20 days. The results for the acetone control beetles reflect previous studies where the SD photoperiod induced diapause in all populations and the LD photoperiod induced diapause only in the Russian population [16, 17]. Indeed the LD photoperiod induced very close to 50 % of beetles to enter diapause in the Russian population. Generally, fecundity was higher and burrowing propensity lower in the LD than SD beetles (Table 1, Figs. 1 and 2).

**Table 1:** The results of a GZLM model testing the effect of population, photoperiod and topical application treatment on a) fecundity and b) burrowing propensity of *L. decemlineata* from three European populations

Effect	Wald *χ* ^2^	df	*p*
a) Fecundity			
Intercept	17055.096	1	<0.001
Weight (covariate)	1066.804	1	<0.001
Population	5661.370	2	<0.001
Treatment	3047.121	2	<0.001
Photoperiod	3215.546	1	<0.001
Population*Treatment	1328.258	4	<0.001
Population*Photoperiod	352.201	2	<0.001
Treatment*Photoperiod	113.182	2	<0.001
Population*Treatment*Photoperiod	429.080	4	<0.001
b) Burrowing propensity			
Intercept	9.270	1	0.002
Population	27.335	2	<0.001
Treatment	10.086	2	0.006
Photoperiod	132.962	1	<0.001
Population*Treatment	1.480	4	0.830
Population*Photoperiod	4.611	2	0.100
Photoperiod*Treatment	0.877	2	0.645

In a) the Akaike Information Criterion (AIC) value of the full model was: 40105.951. Removal of factors and interactions did not improve the model. In b) a model containing all four factors and all interactions had an AIC value of 165.469. Removing sex led to a significant improvement (AIC: 97.530) while in the final model the interaction between Population*Photoperiod*Treatment was also removed (AIC: 93.125). Since the main hypothesis was to test the population*treatment interaction, the two-way interaction effects were left in the final model

In the SD group, JH titers are expected to be low in all populations, and H17 manipulation should have negligible effects. This was observed in both fecundity (Fig. 1c) and burrowing (Fig. 2c) (see Table 1). However, JHA manipulation had a significantly stronger effect on these traits. Fecundity increased in Russian and Italian beetles (Bonferroni Multiple Comparison, BMC: *P* < 0.05) and the proportion burrowed beetles decreased (around 20 %) in all populations (BMC: *P* < 0.001) (Fig. 2c) when treated with the JHA, compared to the acetone control group.

In the LD group, H17 manipulated Russian and Polish beetles had decreased fecundity (BMC: *P* < 0.001) while an opposite effect was observed in the Italian beetles. JHA manipulated beetles had lower burrowing probability compared to those treated with acetone or H17 (BMC: *P* < 0.001, Table 1b) while beetles treated with either acetone or H17 had similar burrowing probability (BMC: *P* =1.000). Importantly, the populations did not differ in receptivity to the applications in terms of burrowing propensity (Table 1b).

## Discussion

The populations originating from different latitudes should show differences in the receptivity to the topical applications of H17 and JHA under the two photoperiods if photoperiodic adaptation has been driven by changes in the endocrinological machinery. We observed that topical applications had different effects on the two measured traits, suggesting that photoperiodic adaptation might not be driven by changes in the endocrinologial machinery. For fecundity we observed different effects among the Colorado potato beetle populations. SD Russian and Polish beetles treated with H17 had decreased fecundity whereas contrary to the expectation, Italian beetles had increased fecundity. One potential explanation for why fecundity did not decrease in all populations might be differences in base JH III titer levels. JH III titers could be higher in the Italian beetles than in the two other populations due to inter-individual variation in natural JH III titer levels, as has been shown previously [18]. Alternatively, the northern populations may have lower JH III levels than the southernmost population since the southernmost population is relatively close to the critical photoperiod also at 12 h light, meaning that a larger proportion of beetles are undergoing reproductive maturation [17] and might have high baseline JH III titers. Consequently, a higher H17 dose would have been needed to elicit a similar decrease in fecundity in Italian beetles as in the northern beetles in the SD photoperiod. Our result that the Italian population reacted to the application of H17 by increasing oviposition supports the conclusion that the populations differ in their hormonal regulation of reproductive output.

The JHA treatment showed more straightforward results regarding fecundity. In the LD photoperiod all populations had increased fecundity after JHA application. However, in the SD photoperiod, both Italian and Russian beetles increased fecundity while no effect was seen in the Polish beetles. We can give two possible explanations, which are linked to the application method, where we used the lowest dose terminating or delaying diapause in a previous study [15, 19]. Firstly, the Polish beetles were heaviest (Poland: 164 ± 2.3 mg, Russia: 160 ± 2.1 mg, Italy: 156 ± 2.0 mg) (univariate ANOVA: F_2,576_ = 4.345, P = 0.013), therefore the applied dose of JHA may have been lower per body mass than in smaller beetles. Secondly, since cuticular thickness generally increases with size in beetles [20, 21], lower amounts of the JHA could have penetrated through the cuticula in larger beetles. Thus, it may be that the large, Polish, individuals received a dose that is below their critical threshold to induce increased fecundity.

In *L. decemlineata*, diapause initiation is strongly related to burrowing into the soil [12]. JHA manipulation resulted in a delayed burrowing in all populations and both photoperiods (data not shown), whereas H17 manipulation resulted in no significant among-population differences in the proportion burrowed beetles in either photoperiod. These results could indicate that burrowing is more strongly under photoperiodic control than fecundity. This is also suggested by observations that unmated female beetles reared in diapause inducing conditions can oviposit small numbers of eggs and still enter diapause, indicating that the reproductive machinery is functioning also during some stage prior to successful diapause initiation [22]. We also find some indication that fecundity and burrowing may be regulated by different hormones. While JH III is known to be involved in both, also ecdysteroids, for instance, may play a role in diapause [13]. In *L. decemlineata* the ecdysteroid titer immediately after adult eclosion is nearly twice as high in beetles destined for diapause than in those that are not destined to enter diapause [19]. The ecdysteroid titer then shows a sex and photoperiod-specific drop and rise again during the 15 days after eclosion. It is unknown whether manipulation of the JH III levels with the JHA or H17 influences the ecdysteroid dynamics in *L. decemlinata*. Nevertheless, it is evident that diapause is regulated by a multifactorial system as suggested by Briers et al. [19].

The main question this study set out to investigate was whether populations show differences in JH III sensitivity which could be linked to a latitudinal gradient. Even though a slight population dependent difference was found in H17 receptivity concerning fecundity, the overall small differences among the populations, particularly regarding diapause related burrowing propensity, indicate that latitudinal patterns seen in life-history and physiological traits [12, 16, 17, 23] are probably not driven by changes in JH III sensitivity. One reason for why latitudinal differentiation is not linked to endocrinological systems could be that, while hormones have large pleiotropic effects, their function can vary depending on life-stage, and therefore, adaptive changes might trade-off against each other in a developmental-stage dependent manner [13]. In *L. decemlineata* JH III is a growth hormone in larvae and controls reproduction and diapause in the adult [13]. Therefore, other, perhaps more likely, candidates driving the observed latitudinal patterns in life-history and physiological traits are genetic changes in the circadian clock, photoperiodic calendar [9, 21, 24, 25] or the insulin signaling pathway, which conveys photoperiodic information to downstream endocrinological elements [10, 13, 26]. Indeed, in a previous study, the circadian genes *period* and *timeless* were found to be differentially expressed in *L. decemlineata* from a northern and a southern European population when reared under similar photoperiods as used in the current study [27]. Another potential among-population difference relates to JH-metabolism. Several studies suggest an important role of JH-esterases (JHE) in regulating JH III-titer in the heamolymph of pre-diapause *L. decemlineata* [12, 13, 28]. In a previous study, however, no difference in expression levels of JHE was found between beetles undergoing pre-diapause development from Russia and Italy [27], indicating that JHE is likely not involved in driving population divergence.

It is important to note that H17 is a mimic of the A-type of allatostatin [29]. While both B- and C-type allatostatin receptors have been found in *L. decemlineata* [30], it is not known whether an A-type receptor is present. In a previous study the A-type receptor was not found in another beetle, *Tribolium castaneum* [31]. However, in the present study manipulation with H17 affected fecundity in the expected manner. This suggests that H17 binds either to an A-type receptor or, if absent, possibly binds with low-affinity to the C-type receptor [30]. Alternatively, H17 may have mediated anti-JH effects documented in the present study through an unknown mode of action. For instance, in a study by Abdel-Latief and Hoffmann, an allatostatin-A derived from the A2b allatostatin precursor in the cockroach *Periplaneta americana* had an allatostatic effect in the beetle *Tenebrio molitor* [32], even though no A-receptor has been found in its close relative, *T. castaneum* [31]. One potential explanation for this effect could be binding of allatostatin-A to other tissues than the corpora allata. For instance, in *Blattella germanica*, allatostatin-A has been shown to influence vitellogenesis by binding directly in the fat body [33]. Therefore, while topical application did affect traits known to be under JH III-mediated control in a logical manner [12, 13, 15], further studies are needed to validate that the topical applications undertaken in the present study truly translate into changed levels of circulating JH III [4, 29].

## Methods

Since JH III has been linked to the decision to enter diapause only in adult beetles, not in larvae, [11, 13], we focused solely on adult beetles in this study. Furthermore, the main sensitive period for photoperiod influencing the decision to enter diapause is the first few days after eclosion to adult [22]. We used descendants of *L. decemlineata* originally collected from potato fields in Russia (61°49'N, 34°10'E), Poland (52°01'N, 20°03'E) and Italy (45°48'N, 12°07'E). Parental generations (minimum 50 families per population and year) were reared and overwintered as described previously [16]. Parent beetles were mated within populations (29 Russian, 20 Polish and 23 Italian families), and checked for deposited eggs daily. Larvae (10 per family) were reared on potato plants till adulthood at 23 °C degrees, 60 % relative humidity and 18 h light. Based on emergence weight (±0.1 mg, AM100; Mettler) adult beetles were divided into treatment groups by ensuring that each family was represented in each treatment group, and the weights were similar among groups. Beetles were kept in pairs of one unrelated female and male in transparent plastic jars (120 ml) containing peat (30 ml). They were reared in two experimental photoperiods: long day (LD, 18 h light) and short day (SD, 12 h light) at 23 °C degrees in environmental chambers (Type B1300; Weiss Technic), and fed *ad libitum* with fresh potato leaves (*Solanum tuberosum*, variety *van Gogh*). The critical photoperiod (when 50 % of adults make the decision to enter diapause) is 17 h for the Russian*,* 15 h and 40 min for the Polish and 15 h and 10 min for the Italian population [17]. Therefore, the short photoperiod is diapause inducing in all populations while the long photoperiod induces diapause only in the Russian beetles [16, 17].

### Topical applications

Haemolymph JH III levels were manipulated by topical applications applied on the ventral thoracic plate at 2 h after lights in the respective cabinet were turned on. Applications were synchronized to control for potential circadian variation in JH III titer or receptivity [34]. To increase JH III levels, 1 μg of the JH III analogue (JHA) pyriproxyfen (Sigma-Aldrich) dissolved in 3 μl acetone was applied once, 1 day after adult eclosion. This dose is the smallest which delays or terminates diapause in *L. decemlineata*, and which incurred no mortality in treated beetles in a previous study [15, 35]. Decreasing natural JH III levels of beetles was done with H17, an allatostatin mimic of the A-type (kindly provided by Prof. Xin-ling Yang, China Agricultural University) shown to have good cuticular penetration in *Diploptera punctata* [29]. We used a concentration of 0.3 nmol H17 in 3 μl of a 1:5 dimethyl sulfoxide (Sigma-Aldrich):acetone solution which was applied 1 day after adult eclosion and every third day thereafter until day 15. The application was repeated because the effect of H17 decreases with time [29], unlike the JHA, which stays potent for at least 15 days [36]. Since diapause induction occurs during the first 10 days of adult life in *L. decemlineata* [12, 15] the manipulation period should cover the induction phase. To control for stress and solute effects, 3 μl of acetone was applied to control beetles every third day until beetles were 12 days old. All beetles were weighed when 6 days old.

### Fecundity and burrowing behaviour

Fecundity and burrowing were used as response indicators to JH level manipulation. Fecundity was measured as the number of hatched eggs. Oviposition was recorded daily until beetles were 30 days old. Eggs from each clutch were counted and maintained on a petri dish, and hatching monitored daily for 10 days. Burrowing into the soil was assessed when beetles were 20 days old.

### Statistical analyses

We used a Poisson generalized linear model (GZLM) with a log-link function to analyze fecundity. Population (Russia, Poland, Italy), photoperiod (short, long) and treatment (JHA, H17, acetone) were included as factors and 6-day weight as a covariate. To analyze the association between sex, population, photoperiod and treatment on burrowing probability, we used a binary logistic GZLM. Model improvement was tracked via the Akaike Information Criterion (AIC). Model AIC values are shown in Table 1. Statistical tests were performed with the IBM SPSS v. 20.0 (IBM SPSS, USA).

## Abbreviations

AIC: Akaike Information Criterion
H17: Allatostatin mimic
BMC: Bonferroni Multiple Comparison
JH: Juvenile hormone III
JHA: Juvenile hormone analog
LD: Long day
GZLM: Poisson generalized linear model
SD: Short day

## References

[1] Gaston K (2003). The structure and dynamics of geographic ranges.

[2] Hendry AP, Farrugia TJ, Kinnison T (2008). Human influences on rates of phenotypic change in wild animal populations. Mol Ecol..

[3] Chevin LM, Lande R, Mace GM (2010). Adaptation, plasticity, and extinction in a changing environment: towards a predictive theory. PLoS Biol..

[4] Zera AJ (2007). Endocrine analysis in evolutionary-developmental studies of insect polymorphism: hormone manipulation versus direct measurement of hormonal regulators. Evol Dev..

[5] Flatt T, Heyland A (2011). Mechanisms of life history evolution. Oxford Biology.

[6] Oostra V, Mateus AR, van der Burg KRL, Piessens T, van Eijk M, Brakefield PM, Beldade P, Zwaan BJ (2014). Ecdysteroid hormones link the juvenile environment to alternative adult life histories in a seasonal insect. Am Nat..

[7] Moran EV, Alexander JM (2014). Evolutionary responses to global change: lessons from invasive species. Ecol Lett..

[8] Nelson RJ, Denlinger DL, Somers DE (2010). Photoperiodism, the biological calendar.

[9] Tauber MJ, Tauber CA, Masaki S (1986). Seasonal adaptations of insects.

[10] Sim C, Denlinger DL (2008). Insulin signaling and FOXO regulate the overwintering diapause of the mosquito *Culex pipiens*. Proc Natl Acad Sci..

[11] Nijhout HF (1994). Insect hormones.

[12] de Kort CAD (1990). Thirty-five years of diapause research with the Colorado potato beetle. Entomol Exp Appl..

[13] Denlinger DL, Yocum GD, Rinehart JP, Gilbert LI (2012). Hormonal control of diapause. Insect Endocrinology.

[14] Khan MA (1988). Brain-controlled synthesis of juvenile hormone in adult insects. Entomol Exp Appl..

[15] Koopmanshap AB, Oouchi H, de Kort CAD (1989). Effects of a juvenile hormone analogue on the eggs, post-embryonic development, metamorphosis and diapause induction of the Colorado potato beetle, *Leptinotarsa decemlineata*. Entomol Exp Appl.

[16] Lehmann P, Lyytinen A, Sinisalo T, Lindström L (2012). Population dependent effects of photoperiod on diapause related physiological traits in an invasive beetle (*Leptinotarsa decemlineata*). J Insect Physiol..

[17] Lehmann P, Lyytinen A, Piiroinen S, Lindström L (2015). Latitudinal differences in diapause related photoperiodic responses of European Colorado potato beetles (*Leptinotarsa decemlineata*). Evol Ecol..

[18] de Kort CAD, Bergot BJ, Schooley DA (1982). The nature and titre of juvenile hormone in the Colorado potato beetle, *Leptinotarsa decemlineata*. J Insect Physiol.

[19] Briers T, Peferoen M, De Loof A (1982). Ecdysteroids and adult diapause in the Colorado potato beetle, *Leptinotarsa decemlineata*. Physiol Entomol.

[20] Evans AR, Sanson GR (2005). Biomechanical properties of insects in relation to insectivory: cuticle thickness as an indicator of insect ‘hardness’ and ‘intractability’. Aust J Zool..

[21] Lease HM, Wolf BO (2010). Exoskeletal chitin scales isometrically with body size in terrestrial insects. J Morphol..

[22] de Wilde J, Duintjer CS, Mook L (1959). Physiology of diapause in the adult Colorado potato beetle (*Leptinotarsa decemlineata* Say) - I the photoperiod as a controlling factor. J Insect Physiol..

[23] Danilevskij AS (1965). Photoperiodism and seasonal development of insects.

[24] Bradshaw WE, Holzapfel CM (2007). Evolution of animal photoperiodism. Annu Rev Ecol Evol Syst..

[25] Pegoraro M, Gesto JS, Kyriacou CP (2014). Tauber E Role for circadian clock genes in seasonal timing: testing the Bünning hypothesis. PLoS Genetics..

[26] Emerson KJ, Bradshaw WE, Holzapfel CM (2010). Microarray reveal early transcriptional events during the termination of larval diapause in natural populations of the mosquito, *Wyeomyia smithii*. PLoS ONE.

[27] Lehmann P, Piiroinen S, Kankare M, Lyytinen A, Paljakka M, Lindström L (2014). Photoperiodic effects on diapause-associated gene expression trajectories in European *Leptinotarsa decemlineata* populations. Insect Mol Biol..

[28] Lefevere KS, Koopmanschap AB, de Kort CAD (1989). Juvenile hormone metabolism during and after diapause in the female Colorado potato beetle, *Leptinotarsa decemlineata*. J Insect Physiol..

[29] Kai Z, Huang J, Tobe SS, Yang X (2009). A potential insect growth regulator: synthesis and bioactivity of an allatostatin mimic. Peptides..

[30] Meng Q, Liu X, Lü F, Fu K, Guo W (2015). Li G Involvement of a putative allatostatin in regulation of juvenile hormone titer and the larval development in *Leptinotarsa decemlineata* (Say). Gene..

[31] Amare A, Sweedler JV (2007). Neuropeptide precursors in *Tribolium castaneum*. Peptides..

[32] Abdel-Latief M, Hoffmann KH (2010). Neuropeptide regulators of juvenile hormone biosynthesis in the beetle, *Tenebrio molitor*. Arch Insect Biochem Physiol..

[33] Martín D, Piulachs MD, Bellés X (1996). Inhibition of vitellogenin production by allatostatin in the German cockroach. Mol Cell Endocrinol.

[34] Zhao Z, Zera AJ (2004). The heamolymph JH titer exhibits a large-amplitude, morph-dependent, diurnal cycle in the wing-polymorphic cricket, *Gryllus firmus*. J Insect Physiol..

[35] Hoffmann EJ, VanderJagt J, Whalon ME (2007). Pyriproxyfen activates reproduction in prediapause northern strain plum curculio (*Conotrachelus nenuphar* Herbst). Pest Manag Sci..

[36] de Kort CAD, Koopmanschap AB, Vermunt AMW (1997). Influence of pyriproxyfen on the expression of haemolymph protein genes in the Colorado potato beetle, *Leptinotarsa decemlineata*. J Insect Physiol.

